# Shared decision-making in Israel: status, barriers, and recommendations

**DOI:** 10.1186/2045-4015-1-5

**Published:** 2012-01-30

**Authors:** Talya Miron-Shatz, Ofra Golan, Mayer Brezis, Gil Siegal, Glen M Doniger

**Affiliations:** 1 Center for Medical Decision Making, Ono Academic Collage, Kiryat Ono, Israel; 2 Wharton School of Business, University of Pennsylvania, Philadelphia, PA, USA; 3 The Unit for Genetic Policy and Bioethics, Gertner Institute for Epidemiology and Health Policy Research, Tel Hashomer, Israel; 4 Center for Clinical Quality and Safety, Hadassah Hebrew University Medical Center, Jerusalem, Israel; 5 Center for Health Law, Bioethics and Health Policy, Ono Academic College, Kiryat Ono, Israel

**Keywords:** Shared decision-making, Israel, patient autonomy, informed consent, health care system, patient participation

## Abstract

Shared decision making (SDM) - involving patients in decisions relevant to their health - has been increasingly influential in medical thought and practice around the world. This paper reviews the current status of SDM in Israel, including efforts to promote SDM in the legislation and healthcare system, its influence in medical training and the national health plans, and funding for SDM-related research. Published studies of SDM in Israel are also reviewed. Although informed consent and patients' right to information are regulated by Israeli law, little provision is made for SDM. Further, there are few organized programs to promote SDM among medical professionals or the public, and governmental support of SDM-related research is minimal. Nonetheless, patients have begun to influence litigation in both formal and informal capacities, medical schools have begun to incorporate courses for improving physician-patient communication into their curricula, and the largest national health plan has initiated a plan to increase public awareness. A review of the limited research literature suggests that although patients and physicians express a desire for greater patient involvement, they often have reservations about its implementation. Research also suggests that despite the positive effects of SDM, such an approach may only infrequently be applied in actual clinical practice. In conclusion, though not actively promoting SDM at present, Israel's universal coverage and small number of health plans make rapid, widespread advances in SDM feasible. Israeli policymakers should thus be encouraged to nurture burgeoning initiatives and set plausible milestones. Comparing the status of SDM in Israel with that in other countries may stimulate further advancement.

## 1. The importance of shared decision making (SDM) in healthcare

Shared decision making (SDM), "the attempt to involve patients in decision-making tasks, especially where decisions, in the face of uncertain or equivocal evidence of benefit, are sensitive to personal preferences" [1], has grown in prevalence worldwide over the last two decades [2]. SDM relates to involving patients in various issues, including screening, treatment options, administration of medication, nutrition, and lifestyle interventions. SDM has influenced the way medicine is practiced and has sparked interest in exploring ways to involve patients in their healthcare decisions and measure the effects of this involvement [3].

Indeed most patients wish to take an active part in choosing among alternative courses of action regarding their health, with the physician either participating in the decision or providing relevant information and then allowing the patient to decide autonomously [4]. Beyond the higher ethical standards associated with greater patient involvement as compared with physician paternalism, SDM has practical merits. An impressive corpus of research has shown that patient involvement leads to better knowledge about treatment options, more realistic expectations concerning disease course and treatment, improved adherence, enhanced patient satisfaction, and sometimes a better clinical outcome [5].

Though ultimately manifest at the level of the patient-physician encounter, SDM must first be legally mandated and medical professionals must be trained to incorporate its principles into their practice. Further, research studies are necessary to monitor its status and drive improvement. Moreover, these activities must be supported and promoted by national health, legal, and other organizations. Some countries have allocated resources for the promotion and evaluation of SDM. In Germany, the ministry of health funded the research consortium ''Patient as partner in medical decision-making'' [6]. In Canada, the importance of SDM is reflected in increased funding for medical training and initiatives to incorporate patient decision aids in medical care [7]. In view of the growing global prevalence and interest in SDM, periodic country-specific status reports facilitate comparison both between and within countries over time. The purpose of the present review is to outline the status of SDM in Israel, a country where universal healthcare coverage and a small number of national health plans make sweeping advances in SDM feasible. Indeed we hope to generate fresh interest in furthering this important approach in Israel.

Portions of this paper (reproduced with permission) have previously been published in abridged form in a special issue of the *German Journal for Evidence and Quality in Healthcare (ZEFQ) *on the global status of SDM [8]. Unlike the *ZEFQ *paper, the present paper makes specific recommendations and provides a more extensive treatment of the relevant topics, as well as the above background on SDM. Further, as the present paper was written for an Israeli audience, there was no need to include background on Israeli demographics, health care spending, and the health care system included in the *ZEFQ *paper.

## 2. Efforts to promote SDM in the Israeli legislation and healthcare system

### 2.1 The micro level: Patient involvement in their own care

Though there is no direct, explicit mandate for SDM in Israeli law, as will be explained below, the requisite conditions are encapsulated in the Patients' Rights Law of 1996. Prior to its passage, support for SDM in Israeli legislation was limited to a clause in the national health insurance law requiring each health plan to furnish a description of its services and make them available to members upon request.

#### Patients' Rights Law

The Patients' Rights Law (hereinafter "the Law") was enacted in 1996 after five private initiatives were combined to form one national proposal. The Law emphasized that patients have rights above and beyond the right to health care alone and was the product of cooperation between members of the Israeli Parliament, government offices, the Association for Civil Rights in Israel, religious and legal representatives, women's organizations and patient and professional associations. The Law defined the rights and obligations of patient-provider relationships, reflecting the shift from a paternalistic model of care to a patient-centered model emphasizing patient autonomy. The main goals of the Law were to define the rights of the patient and protect the patient's dignity and privacy. The Law included rights that were previously recognized in medical ethics and by social and legal norms (e.g., prohibition against discrimination, informed consent, patient access to medical records, privacy of medical information).

The Law relates to medical decision making as an act performed by the physician that may not be executed without the patient's consent. Thus, the Law is mainly concerned with the clinician's duty of disclosure, as required for the patient's informed consent to medical treatment. Yet, these requirements set the necessary conditions for a process of SDM. According to the Law, informed consent must be based on data about the diagnosis and prognosis, the nature of the proposed treatment, expected benefits and likelihood of success, the risks and side effects involved (including pain and discomfort) and those of alternative treatments (whether covered by national health insurance or not) or the lack of any treatment at all. These requirements are the hallmarks of a good decision process characterized by SDM [9]. The clinician is required to "supply the patient medical information to a reasonable extent, so as to enable the patient to decide whether to agree to the treatment proposed" [10]. Thus disclosure according to the Law, must satisfy both what most people in the patient's position would want to know (similar rulings exist in the US, Canada, Australia, and the UK [11-14]) and the needs of the individual patient (similar rulings exist in Germany, Switzerland, and Austria [15]). Several interpretations of the Law suggest that this dual standard for disclosure requires a dialogue with the patient to explore her/his information needs and the extent to which s/he is interested in details about the proposed treatment and its alternatives [16,17].

The Law further states that the clinician shall provide the information to the patient at the earliest stage of the treatment in a manner that maximizes the ability of the patient to understand the information and make a free and independent choice. The consent may be given verbally, in writing, or demonstrated by the patient's behavior. For certain treatments,^a ^informed consent must be given in writing [10]. Notably, the patient's right to refuse treatment is not absolute under the Law. If a patient is in grave danger, the clinician may refer the case to an Ethics Committee for consideration of treatment against the patient's will. In these exceptional situations, the Committee is obliged to listen to the patient and consider whether in the given circumstances, there are reasonable grounds to assume that, after receiving treatment, the patient will give his retroactive consent. The patient's right to access to medical information is similarly limited by clinical privilege (i.e., the clinician may withhold medical information from the patient concerning his medical condition if doing so may be harmful or life-threatening to the patient), again following Ethics Committee approval.

Following enactment of the Law, informed consent became a central issue in malpractice litigation, particularly with regard to adequate communication of the risks associated with a particular treatment, its likelihood of success, and/or the availability of alternative treatments [16]. In 1999 the Israeli Supreme Court handed down a landmark ruling that interference with a patient's right to autonomy is a recognized damage [18]. Thus a patient may be granted dignitary damages for failure to disclose information relevant to the treatment decision even when injury causation or decision causation cannot be proven.

#### The Dying Patient Act

The Dying Patient Act of 2005 (hereinafter "the Act") is consistent with SDM in stipulating that decisions concerning dying patients consider the patient's wishes in addition to the medical condition and degree of suffering. The Act requires that the patient's wishes be periodically reassessed. If the patient is competent at the time of the decision, the determination would be made in accord with the patient's wishes. If the patient is not competent, the physician is instructed to rely upon the patient's previously expressed wishes, either directly or from testimonies of close family and friends.

Autonomous patient requests for active euthanasia or physician-assisted suicide would not be honored under the Act as they are prohibited based upon religious considerations enacted into Israeli law [19]. However, the Israeli Parliament is soon to consider a November 2010 bill that would allow the prescription of lethal drugs to a dying patient upon the patient's request [20]. The bill, modeled upon the Oregon Death with Dignity Act [21], proposes that competent patients be permitted to request the prescription of a lethal drug, provided they are diagnosed with a terminal illness expected to result in death within six months. Still, in keeping with the Act, lethal medication should not be provided immediately upon request, but only after a concerted effort to assess and ameliorate the patient's physical and mental condition [22].

#### Recent initiatives

Israel's population is a heterogeneous mix of immigrants affiliated with three major religions (Judaism, Christianity, Islam) from many different countries, including Ukraine, Ethiopia, Morocco, and the United States. Consequently, there is a diversity of patient beliefs about the degree to which the doctor is the absolute medical authority. Moreover, securing informed consent and access to medical information and services to all Israeli citizens is a significant challenge [23]. The Ministry of Health has pledged to address cultural and language issues associated with provision of services in its 2011-2014 policy plan [24]. Specifically, the Ministry seeks to provide (a) guidance to health services providers regarding the required standard for language and cultural accessibility, (b) training to caregivers in cultural appropriateness, and (c) culturally appropriate materials on health topics, benefits, informed consent, etc. in a variety of languages.

### 2.2 The macro level: Patient involvement in health policy

Public involvement in Israeli health policy has included a variety of formal and informal activities, programs and discussions [25].

#### Formal involvement

Israeli citizens serve on the boards of national health plans and as members of the National Health Council, a nationally representative advisory body to the Ministry of Health [23]. Additionally the Ministry of Health has formed ad hoc committees that include citizen and professional representation. Committees have dealt with such sensitive policy issues as care of terminally ill and dying patients, fertility and procreation, and implementation of a national electronic medical records system [23,26]. Notably, formal bodies seldom include ordinary citizens [23]. For example, only 6 of the 46 members of the National Health Council are non-health professionals, and of these, only two are members suggested by patient advocacy organizations [27].

One prominent committee is the public committee to revise the set of health services benefits to which every Israeli citizen is legally entitled (known in Israel as 'the health basket'). As of this writing, the committee's recommendations regarding technologies to be added to the 'basket', though not legally binding, have been fully adopted. The 'basket' committee is comprised of representatives from the government, the national health plans, and the public. A 2007 government ruling calls for a 16 member committee, four of whom come from the public sector, including experts in ethics [28]. In general, 25% of 'basket' committee members have been ordinary citizens with no medical background [29,30].

In 2003 Israel inaugurated the "Health Parliament" to involve citizens from diverse segments of the population in a deliberative process regarding allocation of public funds for healthcare services. Approximately 130 individuals, randomly selected from all over the country, received extensive orientation to pressing health policy issues from leading experts, and then met to discuss dilemmas associated with equality in health services and prioritization of technologies for funding [31]. Summaries of the proceedings and recommendations of the Health Parliament were presented to the Minister of Health and senior healthcare decision-makers. The initiative was discontinued the following year due to funding problems [32].

#### Informal involvement

There are approximately fifty patient advocacy groups in Israel today, some linked to specific diseases. Members appear at public legal proceedings and are involved in lobbying against government policies that conflict with patient interests. Groups may function individually or in concert with one another, as coordinated by the Israeli Health Consumers' Organization (Z.V.I.) [25] or by a coalition formed by the The Society for Patients' Rights in 2008.

## 3. SDM in Israeli medical training and the national health plans

The deans of all four Israeli medical schools indicated that there were no organized programs to promote SDM at their medical schools or affiliated hospitals. However, courses for improving patient-physician communication have been developed and included in the curricula of most medical schools. At Tel Aviv University, patient empowerment is taught both in formal courses on professionalism and ethics and in simulated role-playing; it is also incorporated into a new 'physician charter' adopted by the Faculty of Medicine. At the Hebrew University, the genetic counseling program offers a course on the psychological aspects of decision-making (developed and taught by the first author) in which future counselors learn about the difficulties in understanding and processing risk information by adopting the perspective of a counselee [33]. The Israel Center for Medical Simulation (MSR), a national, multi-modality, interdisciplinary simulation center based at Sheba Medical Center, offers a wide range of courses designed to improve patient-physician communication skills by exposing students to simulated clinical encounters [34,35].

Key personnel affiliated with the four national health plans in Israel indicated that there were no organized programs to promote SDM among their healthcare providers. However, following a successful pilot [36], the largest health plan ('Clalit') inaugurated a national "Ask Me 3" program to create awareness and reinforce clear health communication, as well as emphasize the patient's role in the medical encounter [37]. The program focuses on three questions: "What is my main problem?", "What do I need to do?", and "Why is it important for me to do this?" A recent study designed to set a baseline for later evaluation of "Ask Me 3" at a major medical center in southern Israel, revealed that 67% of female patients reported asking questions of the medical staff during their hospitalization, but only 33% of patients reported that the staff encouraged such questions [38].

## 4. Research agenda on SDM

In June 1995 the National Health Council designated the Israel National Institute for Health Policy and Health Services Research (NIHP) to oversee implementation of the national health insurance system, conduct relevant research, including surveys, and procure expert professional opinion [39]. The number of SDM-related research studies funded by the NIHP can be taken to reflect the relative importance of SDM to the Israeli research community. Of the 396 NIHP-funded research studies between 1998 and 2010, only 3% were related to SDM. Among these were studies on such topics as engaging psychiatric patients in illness management and the effect of patient participation in improving diabetes management in primary care.

Though the number of studies on a particular topic funded by the NIHP is highly influenced by the number of proposals submitted on that topic and their quality, we believe NIHP should have a clear agenda for evaluating and promoting SDM in Israel, and allocate funds earmarked for this purpose. As a first step, we suggest that the NIHP fund a national project to characterize the status of SDM in Israel, including population data on the prevalence of SDM in clinical practice, subdivided by medical setting. Such a project would also summarize correlates of SDM (e.g., clinical outcomes), as well as barriers to its adoption (see section 6.). Subsequent projects should develop targeted approaches for promoting SDM and assess their efficacy relative to the prior baseline. One such related topic, which could serve as a demonstration project, is prenatal screening, where women are often not fully informed of the meaning of specific screening procedures [40]. We propose that introducing shared decision making to this area might serve to demonstrate the shift incurred in health practices (e.g., avoiding the triple serum screening when one has already decided to undergo amniocentesis), as well as in costs, when patients are invited and equipped to participate in the decision making process.

## 5. Studies of SDM in Israel

In this section, we summarize studies that have investigated SDM in Israel. These studies provide important insights into the factors surrounding sensibilities and issues related to SDM in Israel and thus serve as a context for the development of suitable and effective interventions. We conducted a literature search for relevant articles using Web of Science SCI-EXPANDED, SSCI, and A&HCI databases, EMBASE, PubMed and Google Scholar, with the search terms 'informed consent', 'patient participation' and 'shared decision making', all in conjunction with 'Israel'. We aimed to include papers published after 2000. Additionally, to facilitate a comprehensive and current review, we queried members of the Israeli Social Sciences network, asking them to direct us to relevant works in print or in progress. Though not an exhaustive review of SDM works pertaining to Israel, we believe the following gives a good indication of the type of research being performed, as well as the major trends characterizing the Israeli zeitgeist on SDM.

### Physician advocacy of SDM

Israeli PCPs (*N *= 141) were presented with a vignette describing a hypothetical clinical encounter involving a calm and cooperative or agitated and uncooperative Alzheimer's disease patient and her caregiver [41]. PCPs indicated that they would question, inform, and involve the caregiver (i.e., family), more consistently than the patient, particularly when the patient was agitated. Most PCPs (89%) stated they would reach a decision with the family, 6% stated they would decide paternalistically, and less than 5% stated they would let the family decide autonomously.

SDM is greatly facilitated by the accessibility of medical information on the internet, given that it is directly available to patients. Most Israeli PCPs (82%, *N *= 118; a representative sample) agreed that patient internet use indicates patient involvement and accountability for their medical care. Similarly, 59% expressed satisfaction over patients bringing materials from the internet to the consultation. Regardless, 34% of PCPs felt that the patient or family should rely solely on the physician [42].

#### Patient advocacy of SDM

Israeli, locally representative hospitalized and ambulatory patients (*N *= 274) ranked six issues in terms of priority for improvement [43]. Obtaining more information from the physician and participating in decisions^b ^was ranked most desirable, with 27% ranking it as their top priority and 13% as their second priority. Easier access to specialists or hospital services was ranked next highest, with 18% of patients ranking this top priority and 20% second priority.

Similarly, 613 Israeli hospitalized patients undergoing invasive procedures in various clinical settings were asked about the quality of their informed consent [44]. Though 98% of patients recalled having signed an informed consent, only 39 to 60% recalled receiving explanations about risks of procedures, and 8 to 40% remembered a discussion about alternative management options. Regardless, 80% of patients rated overall satisfaction with the decision making as good or very good, and satisfaction did not correlate with recall of information.

Additionally, 496 of the hospitalized patients and 350 Israeli ambulatory patients indicated their preference for an autonomous, paternalistic, or shared decision-making process [44]. In both settings, approximately 60% of patients preferred SDM, 20% autonomous decision-making, and the remainder paternalistic decision-making.^c^

SDM involves not only physician and patient, but also close family members who may be significantly affected by the consequences of medical decisions^d^. Almost all breast cancer patients interviewed 3-12 months after diagnosis (93%) felt it was important for them to autonomously make medical decisions, but a similar number felt physician (95%) and spousal (89%) concurrence with the treatment decision was important. Interestingly, most patients (88%) and spouses (82%) preferred the physician to make the final decision, possibly reflecting an aversion to the burden borne by the decision-maker, even at the cost of reduced autonomy [45].

In a study investigating perceptions of patient participation in the four national health plans [46], patients (*N *= 656, a nationally representative random sample) did not feel that they were part of the decision-making process in their health plans. Moreover, perception of patient participation was positively correlated with perception of health plan performance.

### SDM in end-of-life care

In a survey on the use of life-sustaining treatments in terminal illness, physician (*N *= 339) and patient (a random sample of 987 elderly Israelis) views were incongruous [47]. Specifically, physicians would order significantly more life-sustaining treatments than patients would want or would order for themselves in the same position. These incongruities may be attributable to cultural norms underlying Israeli medical practice and may be ameliorated by promoting open communication between physicians and patients.

### SDM in actual clinical practice

A qualitative study evaluated whether strategies used by 17 pediatric gastroenterologists to inform adolescents and their families of a diagnosis of irritable bowel syndrome (IBS) and discuss treatment options could be characterized as shared or paternalistic [48]. When interviewed, physicians independently included SDM principles in describing their routine practice. However, observation of the clinical encounters revealed that physicians used tactics to persuade patients to agree with their preferred treatment choice that ultimately reduced patient-physician trust and resulted in low compliance.

Additional evidence for the lack of SDM in actual clinical practice comes from an analysis of 291 videotaped encounters with Israeli PCPs and found that 21% of conflicts related to rationing of health care resources [49]. PCPs most commonly dealt with resource rationing by accepting the situation and withholding alternative treatment options within (appealing rules) or outside of the national health insurance system from their patients.

### Initiatives to promote SDM

PANDEX is a web-based application incorporating decision-analytic methods to assist patients and care providers to reach optimal deliberative decisions [50]. In a pre-clinical feasibility study, Israeli genetic consultants were presented with scenarios of women who had come for genetic consultation, and with PANDEX recommendations for each scenario. Consultants tended to agree with the strategies recommended by PANDEX and acknowledged its capability to provide important insight and serve as a useful tool for patients prior to their meeting with the genetic consultant. Nevertheless, consultants expressed reservations about the integration of a PANDEX-like decision support system in medical care, apparently reflecting their view that consultants play an essential role in both explaining genetic information and facilitating the decision making process.

Recent applications of PANDEX in the prenatal context find that though PANDEX recommended no more than two tests per patient, most patients actually underwent nearly all six of the available tests, and that the PANDEX recommendation was highly influenced by test order [40]. Thus, PANDEX should not serve as the decision-maker, but rather its recommendations should stimulate patient-physician dialogue.

## 6. Barriers to Adoption of SDM

In this section we describe potential barriers to widespread adoption of SDM in Israel. One such barrier is patient and physician ambivalence. As above, although patients indicate a desire for increased involvement, they appear happy with their care and defer difficult decisions to the physician. Similarly, physicians express support for SDM, but often withhold information from patients or apply persuasion tactics so that patients choose the physician's preferred course of action. We believe that this ambivalence, whereby patients and physicians agree with SDM in principle, but are reluctant to incorporate it in practice, represents a significant barrier to the adoption of SDM.

Physician ambivalence may be rooted in another important barrier: lack of formal coursework on SDM in medical education. As a consequence, physicians are untrained in engaging in SDM (even if they desire to do so) and may adopt views inconsistent with SDM. For example, current medical education emphasizes a need for certainty that later compromises the physician's ability to communicate the relative merits of treatment options to patients [51-53]. Also, physicians may avoid disclosure based upon a perception of informed consent as a legal burden. Finally, physicians may interpret the lack of formal coursework as an indication that the medical establishment does not advocate SDM in medical practice.

Another potential barrier pertains to 'collective statistical illiteracy' among physicians and patients [51]. Indeed many physicians are not proficient in interpreting risk and benefit information and will consequently be ill-equipped to explain such information to patients. Patients, as well, are typically not proficient in interpreting such statistics, exacerbating their natural tendency toward reticence in the clinical encounter [54]. Thus rather than discuss information they do not understand, physicians and patients may tend to avoid the type of risk/benefit discourse that characterizes SDM. Moreover, lack of statistical comprehension may lead to a construal of the patient's role as passive rather than informed and participatory [32].

Critically, as indicated above, a nationally-funded population-based study is necessary to firmly evaluate the presence or absence of these barriers in actual clinical practice. Follow-up studies may then systematically evaluate the efficacy of interventions to overcome them. This data can then be used to encourage medical schools and health plans to implement these interventions through such channels as the National Health Council and informal patient advocacy groups.

## 7. Conclusions and policy implications

Indeed, burgeoning initiatives to promote SDM in medical training and practice reflect a growing interest in patient involvement. Only by nurturing these initiatives and with continued support for SDM at multiple levels can efforts to promote SDM be advanced, ultimately resulting in a greater role for citizens in their healthcare and health outcomes. Such advances may serve as a model for furthering SDM in other countries, particularly those with similar national healthcare systems.

This review indicates that Israel possesses the requisite legislative and research infrastructure to facilitate informed patients who are active participants in decisions pertaining to their health: Israel's universal coverage and small number of health plans make rapid, widespread advances in SDM feasible. It is our hope that this review will empower and encourage patient advocacy groups and ultimately policymakers in Israel to advance SDM, aiding them in devising a viable plan with plausibly attainable milestones. Policymakers should focus on funding a population-based project to characterize the status of SDM in Israel, in tandem with engaging and mobilizing medical schools, the national health plans, and practitioners to facilitate the process. Annual audits of the Ministry of Health may be used to track progress.

## Competing interests

The authors declare that they have no competing interests.

## Authors' contributions

TMS conceived of the review, helped search the literature, and contributed to the writing and organization of the manuscript. GMD searched the literature and was primarily responsible for the writing and organization of the manuscript. OG contributed information on the legal aspects of SDM. MB collected and contributed information on SDM in Israeli medical schools and the national health plans. GS investigated and contributed information on research funding for SDM. All of the authors have read and approved the final draft.

## Authors' information

TMS, formerly a researcher at Princeton University, is the founding director of the Center for Medical Decision Making, Ono Academic College, and holds an adjunct position at Wharton School of Business, University of Pennsylvania. OG is a senior researcher at the Unit for Genetic Policy and Bioethics, Gertner Institute for Epidemiology and Health Policy Research and author of the book *Informed Consent to Medical Treatment - The Duty of Disclosure in View of the Patient's Best Interests *(Perlstein-Ginosar Law Books LTD, 2008) [Hebrew]. MB is the director of the Center of Clinical Quality & Safety, Hadassah Hebrew University Medical Center. GS is the director of the Center for Health Law, Bioethics and Health Policy, Ono Academic College, a senior researcher at the Unit for Genetic Policy and Bioethics, Gertner Institute for Epidemiology and Health Policy Research, and a professor of Law with the University of Virginia. GMD is researcher at the Center for Medical Decision Making, Ono Academic College.

## Endnotes

^a^Surgery, other than minor surgery; blood vessel catheterization; dialysis; radiotherapy; in-vitro fertilization; chemotherapy for malignancies. ^b^As participants rated additional information and greater involvement in decision-making as a single item [43], patient preferences for improvement in each of these aspects separately cannot be determined. ^c^These findings are in agreement with those of a study in European patients, of whom 51% favored SDM, 23% autonomous, and the remainder paternalistic decision-making [4]. ^d^Though Israeli law (as in the UK) requires only that the physician involve the patient; no provision is made for close family [55].
